# Distinguishing SARS-CoV-2 Infection and Non-SARS-CoV-2 Viral Infections in Adult Patients through Clinical Score Tools

**DOI:** 10.3390/tropicalmed8010061

**Published:** 2023-01-12

**Authors:** Rujipas Sirijatuphat, Kulprasut Sirianan, Navin Horthongkham, Chulaluk Komoltri, Nasikarn Angkasekwinai

**Affiliations:** 1 Department of Medicine, Faculty of Medicine Siriraj Hospital, Mahidol University, Bangkok 10700, Thailand; rujipas.sir@mahidol.ac.th (R.S.); kulprasut.sir@mahidol.ac.th (K.S.); 2 Department of Microbiology, Faculty of Medicine Siriraj Hospital, Mahidol University, Bangkok 10700, Thailand; navin.hor@mahidol.ac.th; 3 Department of Clinical Epidemiology, Siriraj Medical Research Center, Faculty of Medicine Siriraj Hospital, Mahidol University, Bangkok 10700, Thailand; chulaluk.kom@mahidol.ac.th

**Keywords:** COVID-19, SARS-CoV-2, influenza, respiratory syncytial virus, dengue, chikungunya, zika, predictor, score

## Abstract

This study aimed to determine distinguishing predictors and develop a clinical score to differentiate COVID-19 and common viral infections (influenza, respiratory syncytial virus (RSV), dengue, chikungunya (CKV), and zika (ZKV)). This retrospective study enrolled 549 adults (100 COVID-19, 100 dengue, 100 influenza, 100 RSV, 100 CKV, and 49 ZKV) during the period 2017–2020. CKV and ZKV infections had specific clinical features (i.e., arthralgia and rash); therefore, these diseases were excluded. Multiple binary logistic regression models were fitted to identify significant predictors, and two scores were developed differentiating influenza/RSV from COVID-19 (Flu-RSV/COVID) and dengue from COVID-19 (Dengue/COVID). The five independent predictors of influenza/RSV were age > 50 years, the presence of underlying disease, rhinorrhea, productive sputum, and lymphocyte count < 1000 cell/mm^3^. Likewise, the five independent predictors of dengue were headache, myalgia, no cough, platelet count < 150,000/mm^3^, and lymphocyte count < 1000 cell/mm^3^. The Flu-RSV/COVID score (cut-off value of 4) demonstrated 88% sensitivity and specificity for predicting influenza/RSV (AUROC = 0.94). The Dengue/COVID score (cut-off value of 4) achieved 91% sensitivity and 94% specificity for differentiating dengue and COVID-19 (AUROC = 0.98). The Flu-RSV/COVID and Dengue/COVID scores had a high discriminative ability for differentiating influenza/RSV or dengue infection and COVID-19. The further validation of these scores is needed to ensure their utility in clinical practice.

## 1. Introduction

Coronavirus disease 2019 (COVID-19), caused by the SARS-CoV-2 virus, has spread worldwide since January 2020 [1]. As of 12 December 2022, approximately 4.7 million confirmed cases and 33,392 deaths had been reported in Thailand [2]. The clinical course and severity of COVID-19 vary greatly depending on age, underlying dis-ease, and immune status [3]. The most common clinical presentation of wild-type SARS-CoV-2 infection is a fever, cough, or anosmia, while a stuffy nose, sore throat, and rhinorrhea are reported less frequently [4]. The early diagnosis of COVID-19 is necessary for early treatment and prompt isolation to prevent further transmission [4]. The detection of SARS-CoV-2 RNA by real-time polymerase chain reaction (RT-PCR) from respiratory specimens is the gold-standard test to confirm a diagnosis of COVID-19.

The clinical characteristics of COVID-19 are nonspecific and sometimes difficult to distinguish from other common viral infections that co-circulate in Thailand, such as influenza, respiratory syncytial virus (RSV) infection, dengue fever, chikungunya fever, or zika fever [5,6,7]. Influenza and RSV infection can cause fever, cough, and res-piratory tract symptoms similar to those of COVID-19, whereas dengue fever, chikungunya fever, and zika fever are most commonly associated with acute fever and myalgia [6]. A large sentinel surveillance study in Thailand during the period 2010–2014 (n = 8106) demonstrated that influenza virus and RSV were the most common res-piratory viruses diagnosed among patients with acute fever and cough, accounting for 27% and 6%, respectively [8]. The RSV season significantly overlaps with the influenza season from July to November in Thailand [8]. Dengue virus shows hyper-endemic transmission in Thailand, with an average of 50,000 patients annually [9] and an inci-dence rate of approximately 66 per 100,000 population in 2022. The annual peaks of dengue also occur between June and November in Thailand [10]. Chikungunya virus infection re-emerged in Thailand during the last 10 years, with >27,000 reported pa-tients by the end of 2020 [11] and an incidence rate of approximately 2 per 100,000 population in 2022 [10]. Zika virus infections continued to be reported in Thailand after 2016, with an incidence rate of approximately 0.1 per 100,000 population in 2022 [10]. The co-epidemic or co-infection of these viruses with COVID-19 has been reported [12,13,14,15,16]. The combination of clinical characteristics and initial laboratory investigations could lead to appropriate tests to diagnose suspected cases [17,18,19,20,21,22]. To our knowledge, only few studies have described determining factors or a scoring system to differenti-ate between SARS-CoV-2 infection and non-SARS-CoV-2 viral infections. Our aim was to determine whether COVID-19 and these common viral infections had distinguishing clinical characteristics or basic laboratory parameters, and we intend to develop a predictive model to differentiate COVID-19 and these common viral diseases.

## 2. Materials and Methods

We conducted a retrospective study in adult patients 18 years or older who were diagnosed with COVID-19, influenza, RSV infection, dengue fever, chikungunya fever, and zika fever between September 2017 and April 2020. Eligible patients were obtained from the microbiological laboratory database. The diagnosis of specific diseases was carried out as follows: COVID-19, influenza A or B, and RSV virus were confirmed by the detection of the virus from respiratory specimens through RT-PCR; dengue infection was confirmed by positive nonstructural 1 antigen (NS1Ag) or the detection of dengue viral RNA by RT-PCR; chikungunya infection (CKV) was diagnosed by the detection of CKV viral RNA in blood samples via PCR; and zika virus (ZKV) infection was diagnosed by the detection of ZKV viral RNA in blood or urine samples via PCR. These investigations are the standard diagnostic laboratory tests for the confirmation of SARS-CoV-2 infection and the abovementioned common viral infections in Thailand.

The patients’ medical records were reviewed and collected via standardized record forms that included demographic data; comorbidities; clinical signs and symptoms (fever, body temperature ≥ 37.5 °C, respiratory symptoms, headache, myalgia, arthralgia, rash, and diarrhea); basic laboratory investigations, such as complete blood count (CBC), blood urea nitrogen (BUN), creatinine (Cr), aspartate transaminase (AST), and alanine transaminase (ALT); and the treatment and outcome of the study patients. These basic investigations are the routine laboratory tests for evaluating patients with acute febrile illness in clinical practice. A predictive model based on significant factors comparing COVID-19 to influenza and RSV or dengue was developed and validated.

### 2.1. Statistical Analysis

Descriptive statistics were used to summarize the findings. The mean ± standard deviation (SD) and median (interquartile ranges, IQRs) were used for continuous variables with and without normal distribution, respectively. The diseases were classified into three categories: COVID-19, influenza and RSV, and dengue fever. To compare the qualitative variables between the three categories of infection, the chi-squared test or Fisher’s exact test was used, followed by multiple pairwise comparisons using Bonferroni’s correction. A one-way analysis of variance (ANOVA) or Kruskal–Wallis test was used to compare the quantitative variables between groups. Variables with a univariate *p*-value less than 0.1 were eligible according to the multivariate analysis. Two multiple binary logistic regression models were fitted, i.e., influenza/RSV versus COVID-19 and dengue versus COVID-19, to obtain an adjusted odds ratio (OR) and a 95% confidence interval (CI) of influenza/RSV and dengue. Backward elimination with the likelihood ratio test was applied to obtain statistically significant variables. The calibration of each final logistic model was evaluated using the Hosmer–Lemeshow goodness-of-fit test. A *p*-value > 0.05 indicated a good agreement between the observed and predicted disease.

Discrimination was evaluated using the receiver operating characteristic (ROC) curve and the area under the ROC (AUROC, C statistic) to determine how well the model discriminated between patients with and without a disease. To create the risk score, the regression coefficient (b) of each statistically significant factor was divided by the smallest |b|, and then the values were rounded to the nearest integer. A higher total risk score indicated a higher chance of developing a disease. The accuracy of the total risk score was further evaluated by first creating a score for each patient. This total risk score was then used to create the ROC curve and obtain the AUROC and optimal cutoff point for practical purposes. All statistical data analyses were performed using PASW Statistics 18.0 (SPSS, Inc., 2009, Chicago, IL, USA.). Statistical significance was considered a two-tailed α of 0.05.

### 2.2. Sample Size

The sample size was estimated using a rule of thumb for sample sizes in multiple binary logistic regression analyses [23]. With the expected maximum number of 10 significant factors (i.e., clinical and laboratory parameters for differentiating COVID-19 from other illnesses), 50–100 participants for each disease were required to ensure that the developed model could accurately predict the diagnosis.

## 3. Results

### 3.1. Characteristics of the Study Population

Among the 549 patients enrolled, 100 patients had COVID-19, 100 dengue, 100 in-fluenza, 100 RSV, 100 chikungunya (CKV), and 49 ZKV. The baseline demographic and clinical characteristics of the patients for each disease are shown in Table 1 and Table 2. Females represented approximately 60–80% of all diseases except for COVID-19 (39% female). The mean age for dengue was the lowest (33.5 years), while the mean age of RSV patients was the highest (63.0 years). Comorbidities were found more frequently in influenza and RSV infection (70–76%). Fever was the most common symptom of all dis-eases (63–91%) except ZKV infection (18%). Cough and sore throat were found more often in COVID-19, influenza, and RSV, whereas rhinorrhea, productive sputum, and short-ness of breath were found less often in COVID-19 compared to influenza and RSV. In addition to fever, the predominant symptoms of dengue were myalgia (86%) and head-ache (53%), while those of CKV were arthralgia (78%), myalgia (71%), and rash (65%). All patients diagnosed with ZKV infection had a maculopapular rash. A lower WBC (3355 cells/mm^3^ vs. 4715–8180 cells/mm^3^), lower platelet count (112,500/mm^3^ vs. 185,500–228,500/mm^3^), and higher AST (82 U/L vs. 21–36 U/L) were found in patients with dengue compared to other illnesses.

### 3.2. Comparison of COVID-19 and Influenza/RSV or Dengue

Because CKV and ZKV fever had their own specific clinical features including ar-thralgia or rash, respectively; therefore, we excluded these two diseases and also com-bined influenza and RSV in the same group to compare with COVID-19. The charac-teristics and laboratory parameters of COVID-19 were compared with those of influ-enza/RSV or dengue, as shown in Table 3. Compared to influenza/RSV, COVID-19 pa-tients were younger, less common to have comorbidities, less common to have rhinor-rhea, cough, productive sputum, or shortness of breath, while sore throat was found more frequently in COVID-19. Compared to dengue, COVID-19 patients were older and more likely to have respiratory tract symptoms, while dengue presented more common systemic symptoms, including myalgia, headache, and rash. Furthermore, a lower WBC, absolute lymphocyte count, and platelet count and higher AST or ALT were found more frequently in dengue patients. After dichotomizing the laboratory values for WBC ≥ 4000 cells/mm^3^, absolute lymphocyte count ≥ 1000 cells/mm^3^, platelet count ≥ 150,000/mm^3^, and AST ≥ 40 U/L, all variables remained statistically significant (Table 4).

### 3.3. Model and Score Development

Seven independent variables were entered into a multiple binary logistic regression analysis to identify independent predictors that distinguished influenza/RSV using COVID-19 as a control. Based on backward elimination, five significant factors identified influenza/RSV in opposition to COVID-19, including age > 50 years (OR 3.21, 95% CI 1.24–8.29); the presence of underlying disease (OR 4.16, 95% CI 1.62–10.69); rhinorrhea (OR 11.1, 95% CI 4.08–29.95); productive sputum (OR 23.5, 95% CI 9.38–58.70); and lymphocyte count < 1000 cells/mm^3^ (OR 6.25, 95% CI 2.50–15.72). The model fit the data well based on the Hosmer–Lemeshow goodness-of-fit test (*p* = 0.436) and demonstrated a very good discrimination ability, with an AUROC of 0.944 (95% CI: 0.919, 0.969).

**Figure 1:**
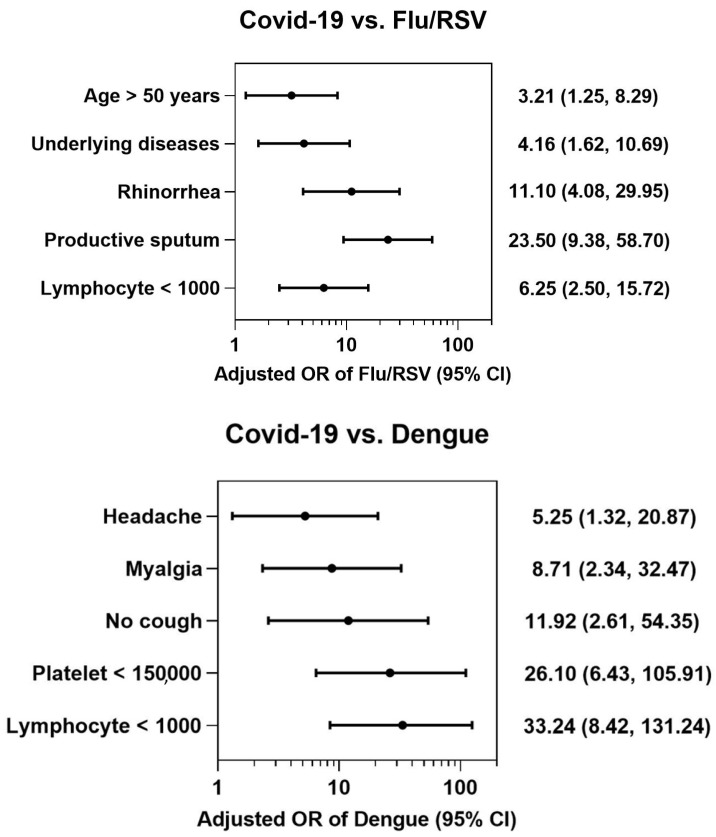
Multivariate logistic regression model of clinical and laboratory parameters for distinguishing between influenza/RSV or dengue and COVID-19. An odds ratio (OR) > 1 is predictive of influenza/RSV or dengue.

Similarly, from the seven independent variables, five significant predictive factors for differentiating dengue from COVID-19 were found in the final model: headache (OR 5.25, 95% CI 1.32–20.87); myalgia (OR 8.71, 95% CI 2.34–32.47); no cough (OR 11.92, 95% CI 2.61–54.35); platelet count < 150,000/mm^3^ (OR 26.1, 95% CI 6.43–105.91); and lymphocyte count < 1000 cells/mm^3^ (OR 33.24, 95% CI 8.42–131.24) (Table 5 and Figure 1). The logistic model for dengue was found to fit the data well (*p* = 0.386 for the goodness-of-fit test) and demonstrated a very high discrimination ability, with an AUROC of 0.979 (95% CI: 0.962, 0.996).

**Figure 2:**
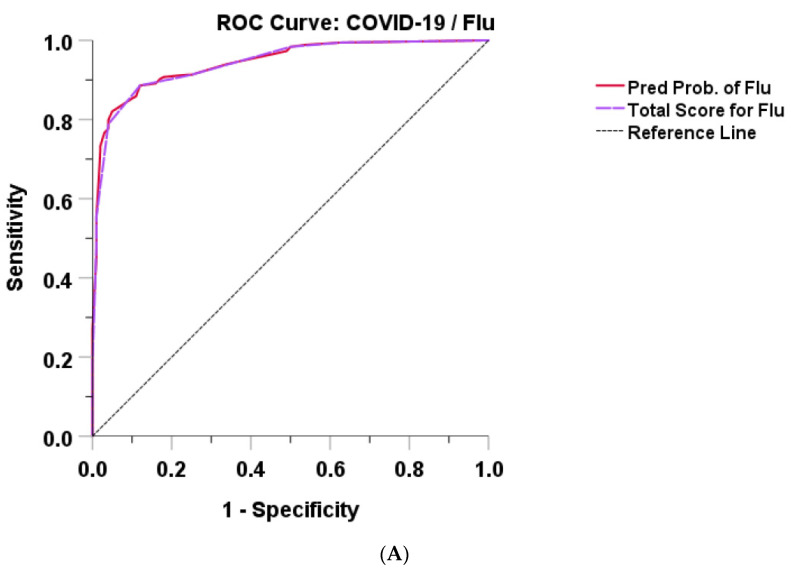

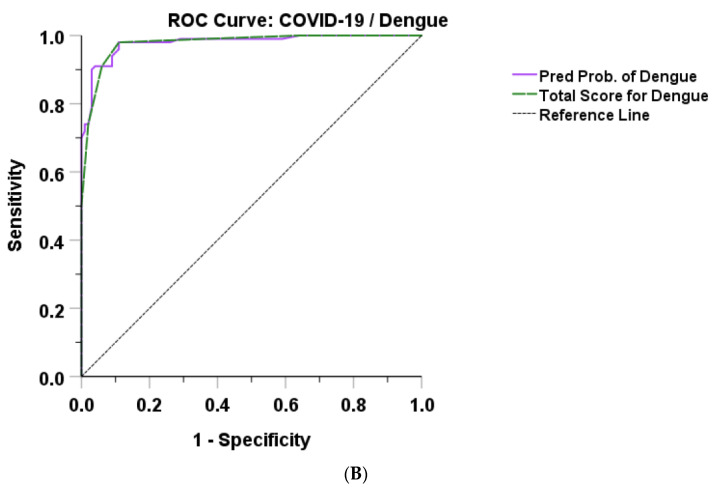
Receiver operating characteristic (ROC) curves of predicted probability and total score for (**A**) influenza or RSV and (**B**) dengue.

Based on the final model, we derived two simplified scores: the Flu-RSV/COVID and Dengue/COVID scores. Each score was weighted by a regression coefficient. For the Flu-RSV/COVID score, the use of five variables (+1 for age > 50 years, +1 for underlying disease, +2 for rhinorrhea, +3 for productive sputum, +2 for lymphocyte count < 1000 cells/mm^3^, and 0 for no indicated factors) provided a total score ranging from 0 to 9 points (Table 6). A higher score indicated a higher chance of being in the influenza or RSV group. For the Dengue/COVID score, the use of five variables (+1 for headache, +1 for myalgia, +1 for no cough, +2 for platelet count < 150,000/mm^3^, +2 for lymphocyte count < 1000 cells/mm^3^, and 0 for no indicated factors) provided a total score ranging from 0 to 7 points (Table 6). A higher score indicated a higher chance of being in the dengue group. Both simplified total scores for influenza/RSV and dengue demonstrated a very good discrimination ability, with an AUROC of 0.942 (95% CI 0.916, 0.968) and 0.977 (95% CI 0.960, 0.995), respectively (Figure 2). The Flu-RSV/COVID score, with a cutoff value of 4, had 88.6% sensitivity and 88.0% specificity for differentiating between influenza/RSV and COVID-19. The dengue/COVID score, with a cutoff value of 4, had 91.0% sensitivity and 94.0% specificity for differentiating between dengue and COVID-19 (Table 7 and Table 8).

## 4. Discussion

The differentiation between COVID-19 and common viral infections co-circulating in tropical regions remains a challenge due to the nonspecific clinical features and limited access to virus-specific diagnostics. It is important to accurately differentiate COVID-19 from these viral illnesses, as their treatment, prognosis, and prevention measures differ. The present study found that rash and arthralgia were hallmark symptoms of ZKV patients and CKV patients, respectively, which was consistent with a previous study [24]. Furthermore, we were able to derive predictive scores with high precision for distinguishing between influenza/RSV or dengue and COVID-19.

Our study found that influenza and RSV patients were older; had more comorbidities; and were more likely to have a cough, fever, productive sputum, rhinorrhea, and shortness of breath, while sore throat was found to be more common in COVID-19 patients. In agreement with previous studies [25], we reported that COVID-19 patients were younger, had fewer comorbidities, were more likely to have a nonproductive cough, and were less likely to have rhinorrhea [26]. Hedberg et al. [27] derived a model that compared COVID-19 with influenza or RSV using demographic, underlying disease, and basic laboratory parameters, achieving moderate accuracy, with an AUROC of 0.75 for influenza and an AUROC of 0.84 for RSV. In our study, we established a model and developed a Flu-RSV/COVID score differentiating between COVID-19 and influenza using five factors, including clinical symptoms and simple laboratory parameters: age > 50 years, the presence of underlying disease, rhinorrhea, productive sputum, and lymphopenia (<1000 cells/mm^3^). With a cutoff value of 4, the score provided high overall sensitivity and specificity (88%) for predicting influenza/RSV. It should be noted that 66% of the influenza patients and 85% of the patients with RSV in our study were admitted to and treated in the hospital, which generally indicated more severe forms of the diseases. On the contrary, the COVID-19 patients treated in the hospital could have presented a mild or severe form of the disease, because early in the pandemic, we admitted all COVID-19 patients to the hospital to prevent further transmission according to the national policy.

The co-existence of COVID-19 and dengue has been reported and is not unusual in dengue endemic areas. COVID-19 patients can present with only a fever and without the respiratory tract symptoms that mimic the clinical signs of dengue, leading to a misdiagnosis of both diseases [12,28]. Furthermore, the antibodies triggered by a SARS-CoV-2 infection can produce rapid false-positive dengue IgG and IgM test results, and vice versa [29]. Previous studies have used only laboratory parameters such as neutrophil count, platelet count, and neutrophil lymphocyte ratio to differentiate between the two diseases [30]. In the present study, we established a model and developed a Dengue/COVID score differentiating between COVID-19 and dengue using five characteristics: headache, myalgia, no cough, thrombocytopenia (platelet count < 150,000/mm^3^) and lymphopenia (lymphocyte count < 1000 cells/mm^3^). With a cutoff value of 4, the score provided a high sensitivity of 91% and a high specificity of 94% for the prediction of dengue. Our findings were partly consistent with a study comparing COVID-19 and dengue, in which a cough and a higher platelet count were found to be suggestive of COVID-19 [31].

Regarding the overall performance of the prediction model, we produced ROC plots that demonstrated a good discriminative ability for both scores (AUROC 0.94, 95% CI 0.92–0.97 for Flu-RSV/COVID score; AUROC 0.98, 95% CI 0.96–0.99 for Dengue/COVID score). In addition, the scores showed high sensitivity and specificity, especially the Dengue/COVID score.

For individual risk prediction in clinical practice, the scores could be used to guide healthcare workers to deliver targeted investigations and appropriate treatments for suspected cases, especially considering that COVID-19 is an endemic disease in tropical regions and the risk of COVID-19 exposure may not be obvious. The use of this study’s scores could be beneficial as an additional tool for the provisional diagnosis of these common viral infections, especially in resource-limited settings where COVID-19-specific diagnosis tests may be unavailable. This study’s scores could also be helpful for physicians when selecting the most rational investigations for the most likely diagnosis, avoiding unnecessary COVID-19 testing. Moreover, a recent study in Thailand demonstrated high IgA and IgM false-positive rates for SARS-CoV-2 antibodies in patients with dengue and other tropical infections, limiting the use of serological assays in the diagnosis of several tropical infections during co-circulation with COVID-19 [32]. As a public health measure, the early suspicion and confirmation of COVID-19 could improve infection control through rapid quarantine or isolation to reduce transmission.

There were some limitations to this study. First, the COVID-19 patients in this study were enrolled from the early-outbreak period in Thailand, which most likely involved the Wuhan strain. During that time, none of the patients received a COVID-19 vaccination. The clinical features are probably different for infections with variants of concern or in patients with partial or complete COVID-19 vaccination; therefore, the model and score require further validation and updating, because the clinical features of COVID-19 have evolved as the COVID-19 situation in Thailand has changed from a pandemic to an endemic. Further research using the 2022 data for patients with COVID-19 and patients with influenza, RSV, and dengue infections should be conducted. Second, there were differences in the data collection intervals for the comparison between SARS-CoV-2 infections and non-SARS-CoV-2 viral infections due to the differences in the duration of the disease outbreaks in Thailand. Third, our results could have been affected by information bias originating from the retrospective review of the medical records, which may have made our results less accurate. Fourth, the generalization of the clinical scores may be difficult, due to the different factors involved in each score, resulting in inconveniences for application in real-life clinical practice.

## 5. Conclusions

Our study identified several independent factors that could help distinguish between SARS-CoV-2 infections and non-SARS-CoV-2 viral infections based on clinical presentation and basic laboratory investigations. The five independent predictors of influenza/RSV were age > 50 years, the presence of underlying disease, rhinorrhea, productive sputum, and lymphocyte count < 1000 cells/mm^3^. Likewise, the five independent predictors of dengue were headache, myalgia, no cough, platelet count < 150,000/mm^3^, and lymphocyte count < 1000 cells/mm^3^. Furthermore, the Flu-RSV/COVID and Dengue/COVID scores demonstrated a good discriminative ability for differentiating between the diseases. Further studies are needed to externally validate the scores for application in co-epidemic situations.

## Figures and Tables

**Table 1:** Characteristics and baseline data of 549 patients with a confirmed diagnosis of COVID-19, influenza, RSV, dengue, chikungunya, or zika virus infection.

	Type of Viral Infection					
	COVID-19	Influenza	RSV	Dengue	Chikungunya	Zika
	(n = 100)	(n = 10)	(n = 100)	(n = 100)	(n = 100)	(n = 49)
Female, n (%)	39 (39)	68 (68)	59 (59)	65 (65)	72 (72)	39 (79.6)
Age (years) ^@^	40.2 ± 15.1	56.7 ± 21.3	63.1 ± 21.3	33.5 ± 13.6	45.1 ± 12.1	42.1 ± 13.7
Body mass index (kg/m^2^) ^@^	24.4 ± 5.2	23.5 ± 7.3	22.8 ± 3.3	23.5 ± 7.0	25.0 ± 4.9	26.06 ± 4.96
Comorbidities, n (%)	26 (26)	71 (71)	76 (76)	29 (29)	36 (36)	16 (32.6)
Diabetes mellitus	10 (10)	31 (31)	20 (20)	9 (9)	13 (13)	4 (8.2)
Hypertension	9 (9)	55 (55)	51 (51)	13 (13)	20 (20)	8 (16.3)
Dyslipidemia	6 (6)	38 (38)	20 (20)	6 (6)	17 (17)	7 (14.3)
Heart disease	2 (2)	33 (33)	26 (26)	5 (5)	2 (2)	1 (2.0)
Lung disease	2 (2)	9 (9)	22 (22)	5 (5)	4 (4)	1 (2.0)
Neurologic disease	3 (3)	19 (19)	20 (20)	5 (5)	4 (4)	0
Liver disease	3 (3)	7 (7)	4 (4)	3 (3)	1 (1)	1 (2.0)
Kidney disease	1 (1)	26 (26)	25 (25)	3 (3)	2 (2)	0
Cancer	3 (3)	14 (14)	23 (23)	1 (1)	4 (4)	3 (6.1)
Setting, n (%)						
Outpatient	0	34 (34)	15 (15)	41 (41)	87 (87)	48 (98)
Inpatient	100 (100)	66 (66)	85 (85)	59 (59)	13 (13)	1 (2)

^@^ Mean ± standard deviation (SD).

**Table 2:** Clinical presentations and laboratory findings by type of viral infection.

	Type of Viral Infection					
	COVID-19	Influenza (n = 100)	RSV	Dengue	Chikungunya (n = 100)	Zika
	(n = 100)		(n = 100)	(n = 100)		(n = 49)
Signs and symptoms, n (%)						
Fever (≥37.5 °C)	77 (77)	83 (83)	72 (72)	91 (91)	63 (63)	9 (18.4)
Rhinorrhea	23 (23)	52 (52)	46 (46)	7 (7)	3 (3)	5 (10.2)
Sore throat	36 (36)	29 (29)	14 (14)	11 (11)	4 (4)	8 (16.3)
Cough	62 (62)	96 (96)	89 (89)	9 (9)	8 (8)	3 (6.1)
Productive sputum	11 (11)	72 (72)	77 (77)	0	2 (2)	0
Shortness of breath	20 (20)	53 (53)	65 (65)	2 (2)	0	0
Diarrhea	9 (9)	2 (2)	24 (24)	13 (13)	2 (2)	0
Myalgia	27 (27)	30 (30)	13 (13)	86 (86)	71 (71)	18 (36.7)
Arthralgia	0	0	0	11 (11)	78 (78)	1 (2.0)
Headache	16 (16)	14 (14)	17 (17)	53 (53)	12 (12)	4 (8.2)
Rash	1 (1)	1 (1)	3 (3)	15 (15)	65 (65)	49 (100)
Laboratory investigation						
Hb (g/dL) ^@^	13.9 ± 1.6	11.5 ± 2.3	10.7 ± 2.2	13.4 ± 1.9	12.8 ± 1.6	13.5 ± 1.3
WBC (cells/mm^3^) ^#^	5120 (3915, 6440)	6640 (4758, 8638)	8180 (4868, 11,868)	3355 (2340, 4863)	4825 (3523, 6215)	4715 (3673, 5473)
Lymphocyte count (cells/mm^3^) ^#^	1602 (1232, 2173)	862 (622, 1256)	986 (499, 1445)	630 (421, 916)	800 (562, 1159)	1301 (911, 1670)
Platelet count (/mm^3^) ^#^	216,500 (173,000, 247,500)	185,500 (147,250, 231,750)	186,500 (131,000, 271,500)	112,500 (66,750, 156,750)	221,000 (170,500, 257,750)	228,500 (201,750, 288, 750)
AST (U/L) ^#^	22 (18, 31)	36 (23, 67)	30 (22, 54)	82 (48, 199)	30 (22, 49)	21 (18, 29)
ALT (U/L) ^#^	24 (16, 37)	25 (15, 38)	24 (14, 42)	56 (30, 134)	27 (17, 42)	16 (11, 25)

^@^ Mean ± standard deviation (SD). ^#^ Median (interquartile range (IQR)).

**Table 3:** COVID-19 vs. common respiratory viruses (influenza and RSV) vs. dengue infection.

	Number (%)			*p*-Value		
	COVID-19 (A) (n = 100)	Influenza and RSV (B) (n = 200)	Dengue (C) (n = 100)	A vs. B vs. C	A vs. B	A vs. C
Female, n (%)	39 (39)	127 (63.5)	65 (65)	<0.001	*	*
Age (years) ^@^	40.2 ± 15.1	59.9 ± 21.5	33.5 ± 13.6	<0.001	*	*
BMI (kg/m^2^) ^@^	24.4 ± 5.2	23.1 ± 5.6	23.5 ± 7.0	0.128	-	-
Comorbidities, n (%)	26 (26)	147 (73.5)	29 (29)	<0.001	*	NS
Diabetes mellitus	10 (10)	51 (25.5)	9 (9)	<0.001	*	NS
Hypertension	9 (9)	106 (53.0)	13 (13)	<0.001	*	NS
Dyslipidemia	6 (6)	58 (29.0)	6 (6)	<0.001	*	NS
Heart disease	2 (2)	59 (29.5)	5 (5)	<0.001	*	NS
Lung disease	2 (2)	31 (15.5)	5 (5)	<0.001	*	NS
Neurologic disease	3 (3)	39 (19.5)	5 (5)	<0.001	*	NS
Liver disease	3 (3)	11 (5.5)	3 (3)	0.523	-	-
Kidney disease	1 (1)	51 (25.5)	3 (3)	<0.001	*	NS
Cancer	3 (3)	37 (18.5)	1 (1)	<0.001	*	NS
Signs and symptoms, n (%)						
Fever (≥37.5 °C)	77 (77)	155 (77.5)	91 (91)	0.011	NS	*
Baseline temperature ^@^	37.3 ± 0.8	38.1 ± 0.9	38.4 ± 1.0	<0.001	*	*
O_2_ sat ^@^	98.0 ± 2.2	94.7 ± 3.7	97.6 ± 1.5	<0.001	*	NS
Rhinorrhea	23 (23)	98 (49.0)	7 (7)	<0.001	*	*
Sore throat	36 (36)	43 (21.5)	11 (11)	<0.001	*	*
Cough	62 (62)	185 (92.5)	9 (9)	<0.001	*	*
Productive sputum	11 (11)	149 (74.5)	0	<0.001	*	*
Shortness of breath	20 (20)	118 (59)	2 (2)	<0.001	*	*
Diarrhea	9 (9)	26 (13)	13 (13)	0.567	-	-
Myalgia	27 (27)	43 (21.5)	86 (86)	<0.001	NS	*
Arthralgia	0	0	11 (11)	<0.001	-	*
Headache	16 (16)	31 (15.5)	53 (53)	<0.001	NS	*
Rash	1 (1)	4 (2)	15 (15)	<0.001	NS	*

* = *p*-value < 0.05, NS = *p* > 0.05. ^@^ Mean ± standard deviation (SD).

**Table 4:** Comparison of initial laboratory investigations.

	Number (%) or Median (IQR)			*p*-Value		
Laboratory Investigation	COVID-19 (A) (n = 100)	Influenza and RSV (B) (n = 200)	Dengue (C) (n = 100)	A vs. B vs. C	A vs. B	A vs. C
Hb (g/dL) ^@^	13.9 ± 1.6	11.1 ± 2.3	13.4 ± 1.9	<0.001	*	NS
WBC (cells/mm^3^) ^#^	5120 (3915, 6440)	7410 (4833, 10,048)	3355 (2340, 4863)	<0.001	*	*
≥4000, n (%)	72 (72)	160/184 (87)	36 (36)	<0.01	*	*
Lymphocyte count (cells/mm^3^) ^#^	1602 (1232, 2173)	904 (562, 1350)	630 (421, 916)	<0.001	*	*
≥1000, n (%)	89 (89)	83/184 (45.1)	20 (20)	<0.01	*	*
Platelet count (/mm^3^) ^#^	216,500 (−173,000, 247,500)	185,500 (−139,250, 245,000)	112,500 (−66,750, 156,750)	<0.001	*	*
≥150,000, n (%)	92 (92)	129/184 (70.1)	28 (28)	<0.01	*	*
AST (U/L) ^#^	22 (18, 31)	32 (22, 58)	82 (48, 199)	<0.001	*	*
≥40, n (%)	18/99 (18.2)	34/92 (37.0)	68/83 (81.9)	<0.01	*	*
ALT (U/L) ^#^	24 (16, 37)	24 (15, 39)	56 (30, 134)	<0.001	NS	*

* = *p*-value < 0.05, NS = *p* > 0.05. ^@^ Mean ± standard deviation (SD). ^#^ Median (interquartile range (IQR)).

**Table 5:** Multiple binary logistic regression analysis of factors differentiating between COVID-19 and influenza/RSV or dengue.

	Risk Factors	b	Adjusted Odds Ratio	95% CI	*p*-Value
COVID-19 vs.	Age > 50 years old	1.168	3.21	1.25–8.29	0.016
Influenza/RSV ^(1)^	Underlying disease	1.425	4.16	1.62–10.69	0.003
	Rhinorrhea	2.403	11.06	4.08–29.95	<0.001
	Productive sputum	3.155	23.47	9.38–58.70	<0.001
	Lymphocyte count <1000 cells/mm^3^	1.836	6.25	2.50–15.72	<0.001
COVID-19 vs.	Headache	1.658	5.25	1.32–20.87	0.019
Dengue ^(2)^	Myalgia	2.165	8.71	2.34–32.47	0.001
	No Cough	2.478	11.92	2.61–54.35	0.001
	Platelet count <150,000/mm^3^	3.262	26.10	6.43–105.91	<0.001
	Lymphocyte count < 1000 cells/mm^3^	3.504	33.24	8.42–131.24	<0.001

B = regression coefficient, adjusted odds ratio (compared to COVID-19). ^(1)^ Variables entered into the model were the abovementioned 5 significant variables, cough, and WBC ≥ 4000 cells/mm^3^. ^(2)^ Variables entered into the model were the abovementioned 5 significant variables, fever, and rhinorrhea.

**Table 6:** Development of score to distinguish COVID-19 and influenza/RSV or dengue.

	Risk Factors	b	b/|Smallest b|	Score
COVID-19 vs.	Age > 50 years old	1.168	1	1
Influenza/RSV	Underlying disease	1.425	1.22	1
	Rhinorrhea	2.403	2.06	2
	Productive sputum	3.155	2.70	3
	Lymphocyte count < 1000 cells/mm^3^	1.836	1.57	2
COVID-19	Headache	1.658	1	1
vs. Dengue	Myalgia	2.165	1.31	1
	No cough	2.478	1.49	1
	Platelet count < 150,000/mm^3^	3.262	1.97	2
	Lymphocyte count < 1000 cells/mm^3^	3.504	2.11	2

b = regression coefficient.

**Table 7:** Accuracy of total score for influenza/RSV and dengue.

	Number (%)			Number (%)	
Score for Influenza	COVID-19 (n = 100)	Influenza (n = 184)	Score for Dengue	COVID-19 (n = 100)	Dengue (n = 100)
0	37 (37)	1 (0.5)	0	36 (36)	0 (0)
1	13 (13)	2 (1.1)	1	28 (28)	1 (1)
2	25 (25)	13 (7.1)	2	25 (25)	1 (1)
3	13 (13)	5 (2.7)	3	5 (5)	7 (7)
4	8 (8)	18 (9.8)	4	4 (4)	17 (17)
5	3 (3)	43 (23.4)	5	2 (2)	24 (24)
6	0	14 (7.6)	6	0 (0)	28 (28)
7	1 (1)	54 (29.3)	7	0 (0)	22 (22)
8	0	8 (4.3)			
9	0	26 (14.1)			

**Table 8:** Sensitivity and specificity of different cutoff points for differentiating between influenza/RSV or dengue and COVID-19.

Score for Influenza/RSV: Cutoff Point	Sensitivity (95% CI)	Specificity (95% CI)
≥3	91.3 (86.3, 95.0)	75.0 (65.3, 83.1)
≥4	88.6 (83.1, 92.8)	88.0 (80.0, 93.6)
≥5	78.8 (72.2, 84.5)	96.0 (90.1, 98.9)
Score for Dengue: Cutoff Point	Sensitivity (95% CI)	Specificity (95% CI)
≥3	98.0 (93.0, 99.8)	89.0 (81.2, 94.4)
≥4	91.0 (83.6, 95.8)	94.0 (87.4, 97.8)
≥5	74.0 (64.3, 82.3)	98.0 (93.0, 99.8)

## Data Availability

Not applicable.
