# Correction: Sirijatuphat et al. Distinguishing SARS-CoV-2 Infection and Non-SARS-CoV-2 Viral Infections in Adult Patients through Clinical Score Tools. *Trop. Med. Infect. Dis.* 2023, *8*, 61

**DOI:** 10.3390/tropicalmed10120341

**Published:** 2025-12-05

**Authors:** Rujipas Sirijatuphat, Kulprasut Sirianan, Navin Horthongkham, Chulaluk Komoltri, Nasikarn Angkasekwinai

**Affiliations:** 1 Department of Medicine, Faculty of Medicine Siriraj Hospital, Mahidol University, Bangkok 10700, Thailand; 2 Department of Microbiology, Faculty of Medicine Siriraj Hospital, Mahidol University, Bangkok 10700, Thailand; 3 Department of Clinical Epidemiology, Siriraj Medical Research Center, Faculty of Medicine Siriraj Hospital, Mahidol University, Bangkok 10700, Thailand

There was an error in the original publication [1] in the first paragraph of Material and Methods. 

In the original sentence ‘We conducted a retrospective study in adult patients 18 years or older who were diagnosed with COVID-19, influenza, RSV infection, dengue fever, chikungunya fever, and zika fever between September 2017 and July 2020’, the period was incorrectly stated as ‘between September 2017 and July 2020’. The corrected text is as follows:

“We conducted a retrospective study in adult patients 18 years or older who were diagnosed with COVID-19, influenza, RSV infection, dengue fever, chikungunya fever, and zika fever between September 2017 and April 2020.”

The authors apologize for any inconvenience caused and state that the scientific conclusions are unaffected. This correction has been approved by the Academic Editor, and the original publication has also been updated.
